# Reviewing Magnetic Particle Preparation: Exploring the Viability in Biosensing

**DOI:** 10.3390/s20164596

**Published:** 2020-08-16

**Authors:** Daniel Kappe, Laila Bondzio, Joris Swager, Andreas Becker, Björn Büker, Inga Ennen, Christian Schröder, Andreas Hütten

**Affiliations:** 1 Faculty of Physics, Bielefeld University, P.O. Box 100131, 33501 Bielefeld, Germany; dkappe@physik.uni-bielefeld.de (D.K.); laila.bondzio@uni-bielefeld.de (L.B.); Joris.Swager@gmx.de (J.S.); andreas.becker@uni-bielefeld.de (A.B.); bjoern.bueker@uni-bielefeld.de (B.B.); ennen@physik.uni-bielefeld.de (I.E.); christian.schroeder@fh-bielefeld.de (C.S.); 2 Bielefeld Institute for Applied Materials Research, Computational Materials Science and Engineering, University of Applied Sciences Bielefeld, Wilhelm-Bertelsmann-Str. 10, 33602 Bielefeld, Germany

**Keywords:** magnetic nanoparticle properties, Heusler phases, e-beam lithography, magnetic ratchets

## Abstract

In this review article, we conceptually investigated the requirements of magnetic nanoparticles for their application in biosensing and related them to example systems of our thin-film portfolio. Analyzing intrinsic magnetic properties of different magnetic phases, the size range of the magnetic particles was determined, which is of potential interest for biosensor technology. Different e-beam lithography strategies are utilized to identify possible ways to realize small magnetic particles targeting this size range. Three different particle systems from 500 μm to 50 nm are produced for this purpose, aiming at tunable, vertically magnetized synthetic antiferromagnets, martensitic transformation in a single elliptical, disc-shaped Heusler Ni_50_Mn_32.5_Ga_17.5_ particle and nanocylinders of Co_2_MnSi-Heusler compound. Perspectively, new applications for these particle systems in combination with microfluidics are addressed. Using the concept of a magnetic on–off ratchet, the most suitable particle system of these three materials is validated with respect to magnetically-driven transport in a microfluidic channel. In addition, options are also discussed for improving the magnetic ratchet for larger particles.

## 1. Introduction

Over the last 30 years, magnetically-driven transport and the detection of biomolecular interactions has matured into a fascinating and vibrantly developed field of science, motivated and accelerated by two scientific challenges. The first challenge is to create a lab-on-chip platform purely based on magnetism and to explore and expand its ability to transport, separate and detect biomolecule-loaded magnetic carriers in a controlled manner. The second challenge, which also leaves much room for future research, is to use this steadily increasing efficiency of control and detection to track and analyze the dynamics of biomolecular processes in situ.

Magnetic labelling of biomolecules and their separation from unlabeled species is a common method in molecular biology, e.g., the MagneSphere™ technology with 1 µm paramagnetic particles developed by Promega (Madison, WI, USA) for mRNA purification in the early 1990s. However, the simultaneously discovered giant magnetoresistance (GMR) in 1988 [1,2] allows one to sensitively detect small magnetic fields and huge interest was raised for analytical refinement and highly sensitive quantification of magnetically labeled biomolecules in vitro and ex vivo. Realizing and exploring this potential for biology, Baselt et al. [3] presented the first GMR biosensor in 1998, and their potential down to the single-molecule limit was investigated and compared to, e.g., fluorescence techniques [4,5,6,7,8,9,10,11]. Nowadays, immunoassays are established in life science to determine the concentration of macromolecular analytes (e.g., antigens) in solution [8,9]. In particular, spintronics and its application inspire a new class of biomedical diagnostic devices, suitable for benchtop bioassays as well as for point-of-care [10,11,12]. In parallel with this research, intensive studies on magnetic nanoparticles and their sensing have been conducted [13,14,15]. Moreover, novel concepts in nanomagnetism such as vortex structures [16], magnetic skyrmion configurations [17] and giant Anomalous Hall Effect [18,19] have attracted a great deal of attention, because they promise highly sensitive, linear and hysteresis-free sensing systems. In addition to the Anomalous Hall Effect, its planar counterpart [20] has recently enabled a very sensitive detection of magnetic stray fields down to the level of single magnetic nanoparticles.

Magnetically-driven transport has been advanced over the last decade utilizing concepts such as Brownian magnetic ratchet [21,22] and magnetic patterning of planar as well as of rolled-up structures [23,24].

Similarly, producing and handling nm- to µm-scale magnetic particles has been brought to a high degree of perfection in the last few years [25,26,27]. The surface functionalization and the specific attachment of biomolecules to magnetic particles have also advanced over the last years by state-of-the-art surface chemistry and biochemical linking systems [28].

Recently, we have suggested studying protein synthesis in microfluidic environments using tailored superparamagnetic particles [29]. Superparamagnetic nanoparticles are characterized by the fact that they have no remanence without the influence of an external magnetic field and thus do not exhibit a magnetic stray field. Only in the presence of an external magnetic field do nanoparticles exhibit ferromagnetic properties. The envisioned device should combine lysis of a selected single cell, labelling of selected polysomes, separation, detection, and quantification. The realization of such novel approaches requires four developments: (i) production of superparamagnetic particles of sufficiently small size matching that of the relevant biomolecules, (ii) their functionalization for specific binding to the biomolecule of interest, (iii) the separation in microfluidic devices, and (iv) the detection and analysis of the isolated structures. 

Regarding these requirements and the scope of this special issue, we want to focus on three aspects. Firstly, based upon literature data of different magnetic phases, we determine the size range of the magnetic particles, which is of potential interest for biosensor technology. Secondly, via a top-down approach, we utilized different e-beam lithography strategies to identify possible ways in realizing small magnetic particles targeting this size range. Hence, three different particle systems from 500 μm to 50 nm are produced for this purpose, aiming at tunable, vertically magnetized synthetic antiferromagnets, martensitic transformation in a single elliptical, disc-shaped Heusler Ni_50_Mn_32.5_Ga_17.5_ particles and nanocylinders of the Co_2_MnSi-Heusler compound. Perspectively, new applications for these particle systems in combination with microfluidics are briefly addressed. Thirdly, using the concept of a magnetic on–off ratchet, the most suitable particle system of these three materials is validated with respect to magnetically-driven transport in a microfluidic channel confirming the viability of these particle systems for directed magnetic transport, which is an essential part of modern biosensing and separation techniques. In addition, options are also discussed for improving the magnetic ratchet for larger particles.

## 2. Intrinsic Magnetic Properties as Decision Support

**Figure 1:**
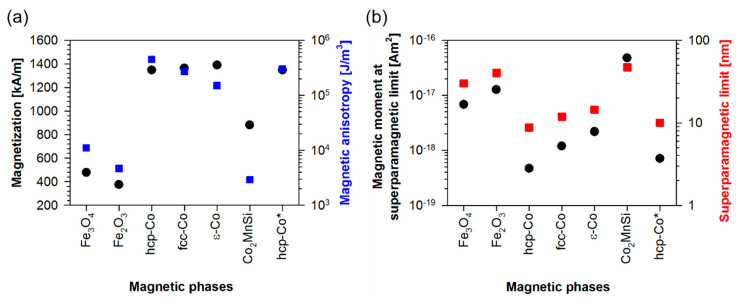
Intrinsic magnetic data of different phases taken from [30] for Fe_2_O_3_ and Fe_3_O_4_, from [31,32] for hcp-Co, fcc-Co and ε-Co and from [33] for Co_2_MnSi. The hcp-Co phase marked by stars have magnetocrystalline anisotropies reduces by a factor of 1.5 due to grain size refinement of the underlying microstructure as is reported in [34]. (**a**) Comparison between saturation magnetization (black filled circles) and magnetocrystalline anisotropy (blue filled squares) at room temperature and; (**b**) Resulting superparamagnetic limits (red filled squares) calculated employing Equation (1) and the calculated magnetic moment at the superparamagnetic limits (black filled circles) considering spherical nanoparticles.

Concerning the synthesis of very small magnetic nanoparticles, the first question that arises is which magnetic material classes have the potential for biotechnological applications based on their intrinsic magnetic properties. From a magnetic point of view, the key requirements for magnetic material classes for biosensing applications are the following properties: (1) a large superparamagnetic limit, which marks the transition between superparamagnetism and ferromagnetism, (2) a very large susceptibility, which makes it possible to reach saturation magnetization at very small external magnetic fields and (3) a high magnetic moment, which realizes the transport of the corresponding nanoparticles in magnetic potential landscapes. Figure 1 compares oxide and metallic phases. The two oxide phases Fe_2_O_3_ and Fe_3_O_4_ are characterized by relatively small magnetizations 375 kA/m and 478 kA/m, respectively, but also by small magnetocrystalline anisotropies, see Figure 1a. The last property is the key for calculating the corresponding superparamagnetic limit. Considering that the nanoparticles are to be stable in this state over a period of one year, Equation (1) provides the corresponding superparamagnetic limit:

(1) $$L_{spm}={\left(\frac{228Tk_{B}}{\pi K_{A}}\right)}^{\frac{1}{3}},$$

*K_A_* being the magnetocrystalline anisotropy, *T* the temperature of 300 K and *k_B_* = 1.380649 × 10^−23^ J/K the Boltzmann constant. 

Thus, the resulting superparamagnetic limits are about 40 nm for Fe_2_O_3_ and about 30 nm for Fe_3_O_4_. Magnetite nanoparticles are the success model in biosensor technology, as they are proven to be biocompatible and have paved the way to magnetic beads [35]. If superparamagnetic magnetite nanoparticles are encapsulated within a spherical polymer carrier matrix with a sufficiently large distance and hence no dipolar magnetic interactions between them, the magnetic moment of the bead can be freely tuned as a function of the number of these magnetite particles within each polymer sphere. Overall, however, the resulting behavior of the bead remains superparamagnetic. These beads are commercially available under the brand name MyOne when the bead diameter is 1 μm. Different beads can be purchased in the size range between 100 nm and 3 μm.

The three Co-phases, on the other hand, show comparatively high values for both the saturation magnetization and the magnetocrystalline anisotropy. This limits their superparamagnetic limit to values of 8.7 nm for hcp-Co, 11.9 nm for fcc-Co and 14.4 nm for ε-Co. Experiments with high-energy ball milling at least for hcp- and fcc-Co [34] show that the magnetocrystalline anisotropy is reduced by a factor of 1.5 due to the associated grain size refinement. This increases the superparamagnetic limit of hcp-Co only insignificantly as shown in Figure 1b.

Co_2_MnSi as a representative of the material class of Heusler alloys, causing a sensation as spintronic materials [36], characterized by a very low magnetocrystalline anisotropy and a saturation magnetization between the oxide- and the Co-phases, see Figure 1a. This leads to a superparamagnetic limit of 47 nm and to the largest magnetic moment when comparing all different materials in Figure 1b. Due to these magnetic properties, Heusler alloys are interesting for biosensing. From our experience, Co_2_MnSi can also be prepared very well in the form of thin magnetic films by sputtering.

**Figure 2:**
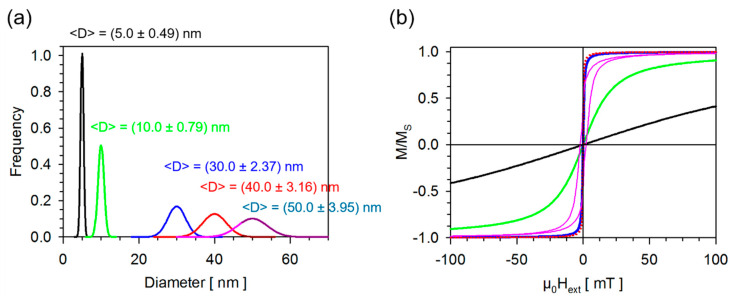
Hysteresis curves of Co_2_MnSi calculated applying Equation (2), assuming the nanoparticle size distributions provided in (**a**) to monitor the transition from purely superparamagnetic behavior towards partly ferromagnetic behavior. Associated curves in (**a**,**b**) have the same color code. (**a**) Underlying nanoparticle size distributions of Co_2_MnSi color-coded, characterized by their mean size and assumed standard deviation; (**b**) The resulting magnetization reversal of each nanoparticle distribution is calculated using Equation (2) considering a mixture of partly superparamagnetic particles and partly ferromagnetic ones. The transition between pure superparamagnetic towards ferromagnetic behavior will not occur until the superparamagnetic limit of Co_2_MnSi. Thus, it is reflected in the visible coercivity. Nanoparticles with diameters of 30 nm and beyond show a very large susceptibility.

**Figure 3:**
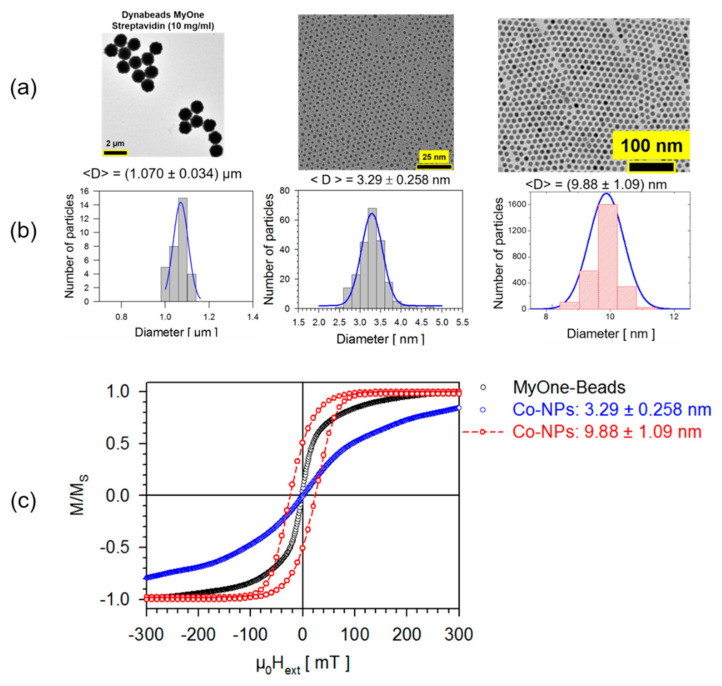
Comparison between hysteresis curves of MyOne beads and Cobalt nanoparticles synthesized via thermolysis [38]. (**a**) Microstructure of each species taken by transmission electron microscopy (TEM); (**b**) The corresponding particle size distribution derived from the TEM analyses; (**c**) Normalized magnetization reversals of each species.

The Heusler alloy is also superior in terms of susceptibility, which is documented in Figure 2. Visualizing the magnetization reversal of Co_2_MnSi based on underlying nanoparticle distributions reveal that nanoparticles at or below their superparamagnetic limit are at the same time not characterized by a decent susceptibility unless they are about 30 nm in diameter. This can been seen also in measurements presented in Figure 3.

The magnetization reversals compared in Figure 2 are calculated applying Equation (2):

(2) $$\begin{array}{l} \begin{array}{cc} M(H_{ext}) & =\left\{\int_{L_{spm}}^{b}\frac{1}{\sigma \sqrt{2\pi }}\exp\left(-\frac{{\left(D-\mu \right)}^{2}}{2\sigma^{2}}\right)\frac{4}{3}\pi {\left(\frac{D}{2}\right)}^{3}dD\right\}\;\cdot \\ & \left(\frac{2}{\pi }\mathrm{atan}\left(\frac{H_{ext}\pm H_{C}}{H_{C}}\tan\left(\frac{\pi S}{2}\right)\right)\right)+\\ & \left\{\int_{a}^{L_{spm}}\frac{1}{\sigma \sqrt{2\pi }}\exp\left(-\frac{{\left(D-\mu \right)}^{2}}{2\sigma^{2}}\right)\frac{4}{3}\pi {\left(\frac{D}{2}\right)}^{3}dD\right\}\cdot \\ & \int_{a}^{L_{spm}}\left(\frac{1}{\sigma \sqrt{2\pi }}\exp\left(-\frac{{\left(D-\mu \right)}^{2}}{2\sigma^{2}}\right)\coth\left(\frac{\frac{4}{3}\pi {\left(\frac{D}{2}\right)}^{3}MH_{ext}}{k_{B}T}\right)-\frac{k_{B}T}{\frac{4}{3}\pi {\left(\frac{D}{2}\right)}^{3}MH_{ext}}\right)\;dD \end{array}\\ \mathrm{with}\;H_{C}=0.482\frac{2K}{M}\;\mathrm{and}\;S=0.5 \end{array}$$

In red is the ferromagnetic contribution, which is weighted by the volume fraction of the ferromagnetic nanoparticles written in braces. The model for the ferromagnetic hysteresis is taken from [37] and contains two parameters. Firstly, the coercivity is provided by the anisotropy field of a statistically oriented magnetic particle ensemble. The anisotropy field itself is defined as the ratio of twice the magnetocrystalline anisotropy constant of Co_2_MnSi divided by the saturation magnetization at 300 K taken from Figure 1. The sign of the coercivity depends on the direction of the external field and is positive when ramping the external field from the positive filed maximum towards the negative field minimum. It is negative when ramping the external field from the negative field minimum towards the positive field maximum. *S* is the squareness of the ferromagnetic loop describing the ratio of remanence to saturation magnetization. The superparamagnetic contribution, in blue, is a Langevin equation that is weighted by the volume fraction of the superparamagnetic nanoparticles written in braces. *M* denotes the saturation magnetization, *K* the magnetocrystalline anisotropy and *k_B_* is the Boltzmann constant. *H_ext_* in both parts is the applied external magnetic field.

D is the diameter of each nanoparticle within the distribution, *μ* the mean diameter and *σ* the standard deviation of the nanoparticle size distribution. It should be explicitly pointed out that this description of the magnetization reversal does not take into account dipolar interactions between the nanoparticles and therefore the presence of the coercivity is solely due to the presence of ferromagnetic nanoparticles.

The fact that small superparamagnetic nanoparticles will show a magnetization reversal with a moderate susceptibility only can be observed when measuring the hysteresis of MyOne beads, compare Figure 3c. MyOne beads have an exceptionally sharp particle size distribution with a standard deviation of only 3.2%. The missing coercivity clearly indicates, firstly, that the Magnetite nanoparticles used as superparamagnetic filling are below their superparamagnetic limit and, secondly, that there are indeed no dipolar interactions between these nanoparticles. This situation changes when comparing with Cobalt nanoparticles prepared via thermolysis [38]. A closed packed monolayer of Co nanoparticle is ensembled, see Figure 3, with a size distribution around their superparamagnetic limit clearly exhibiting a pronounced hysteresis, which can be caused by two effects. The first one being the superparamagnetic limit and the second is the dipolar interparticle interactions. The resulting susceptibility of very small Co-nanoparticles is rather weak. Nevertheless, the magnetic nanoparticles and beads put together in Figure 3 are an example of isolated magnetic objects that would be suitable for biosensor applications. In many cases, an extremely sophisticated synthesis process using various chemical methods can realize magnetic nanoparticles, but they are still present in the form of agglomerates and would hardly be suitable for biosensor applications.

As a conclusion of the discussion so far, it can be said that magnetic nanoparticles based on Heusler alloys, which have already proved their relevance as magnetic electrodes in spintronics, have superior magnetic properties in comparison to all other presented magnetic phases and hence are an interesting class of materials for biosensing and could be used as magnetic markers. From the point of view of the target size, these nanoparticles should ideally be distinguished by a narrow size distribution with an average value around 30 nm.

## 3. Top-Down Approaches for Realizing Nanoparticles from μm towards nm

### 3.1. Tunable Perpendicularly Magnetized Synthetic Antiferromagnets

The top-down approach is based on multilayered systems, which are then manufactured into micro- or nanostructures using e-beam lithography strategies. In contrast to the alternative, the bottom-up approach in which nanoparticles are synthesized by combining atoms via chemical means, the top-down approach has the benefit of integrating complex magnetic interactions in the multilayer systems and thus also in the resulting nanostructures. On the other hand, especially for ternary or quaternary alloy systems, the composition responsible for the physical properties can be transferred very precisely into the resulting micro- or nanostructures when starting from multilayer systems. In this section, we want to show this by means of three selected examples from our work and thus identify possible applications for biosensor technology.

The first example is based on a very elegant idea by Cowburn et al. [39,40], the realization of tunable perpendicularly magnetized synthetic antiferromagnets. In the range of small external magnetic fields ΔB_ext_, the interlayer exchange coupling integrated into the underlying multilayer system ensures antiparallel alignment of adjacent magnetic layers and thus complete compensation of the overall magnetization. Thus, as in the case of superparamagnetic nanoparticles, in the ΔB_ext_ region, these layered systems have no magnetic stray field. If the external magnetic fields exceed the range of ΔB_ext_, the layered system becomes abruptly saturated.

**Figure 4:**
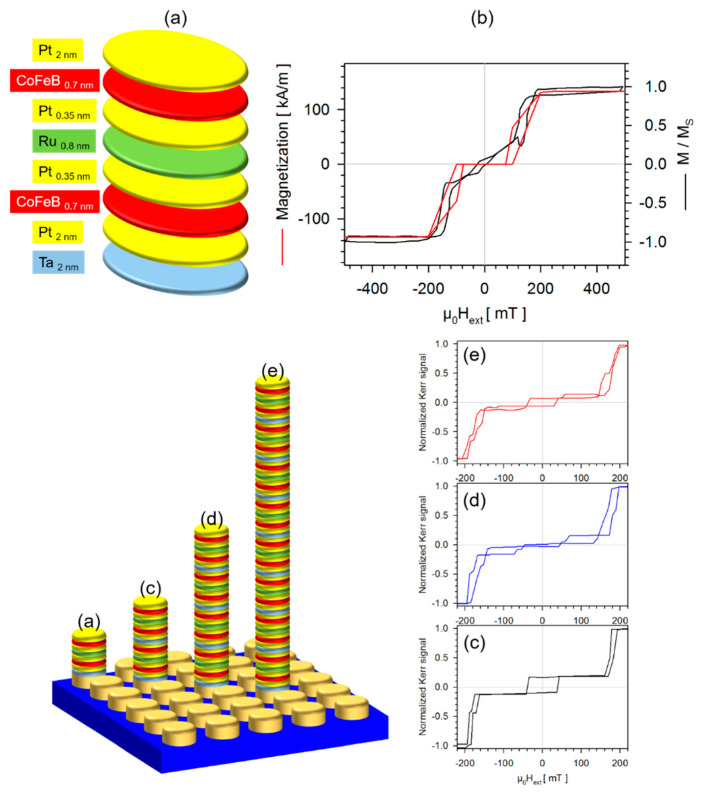
Switching behavior of perpendicularly magnetized synthetic antiferromagnets. (**a**) Basic motif of the layer stack provided with layer thickness. The diameter of the discs are 5 μm; (**b**) Comparison of the magnetization reversal of particles of the basic motif. In black the normalized magnetization of particles suspended in DI water. The measurements were performed by an alternating gradient magnetometer. In red, a micromagnetic calculation of particles still being arranged in the array. The quasistatic micromagnetic simulation package MicroMagus version 7.1 was employed to do these calculations; (**lower left**) Schematic presentation of a disc array fabricated employing e-beam lithography: positive resist (AR-P 5350) was dripped on a Si/SiO_2_ (50 nm) wafer piece and evenly distributed via spin coating (200 rpm for 1 s and 5000 rpm for 30 s). After a backing step at 100 °C for 4 min, e-beam lithography was performed to expose square arrays of pillars, each with 5 μm in diameter and 10 μm apart from each other. After exposure and development (developer: AR-300-35, approx. 24 s) only the resist pillars remained. The multilayered stacks were then magnetron sputtered onto these resist pillars: (**a**) the basic motif, (**c**) twice the basic motif, (**d**) 4 times of repetitions of the basic motif and (**e**) 8 times of repetitions of the basic motif. The resist was dissolved with ethanol and particles were suspended in DI water; (**lower right**) Corresponding magnetization reversal determined by polar Moke of all layer stacks (**c**–**e**) when still being fixed in arrays onto their pillars.

In Figure 4, the development of this switching behavior is summarized as a function of the repetition of the basic motif of the multilayered systems. The Pt-layers interfaces sandwiching the Ru layers within the layer stacks, visualized in Figure 4, play a central role. They simultaneously fulfil two tasks; firstly, the stabilization of the perpendicular magnetic anisotropy, and secondly, the damping of the interlayer exchange coupling. To determine the optimum layer thickness of each layer of the basic motif, firstly, the layer thickness of the two CoFeB/Pt-pairs was varied with a fixed Ru-layer thickness at 0.5 nm aiming for the largest ΔB_ext_ value. This was reached at a CoFeB layer thickness of 0.7 nm and a Pt layer thickness of 0.35 nm. In the second step, to optimize the slope of the switching from the compensated to the ferromagnetic state, the Ru-layer thickness was varied between 0.3 nm to 0.9 nm with a fixed CoFeB layer thickness of 0.7 nm and a fixed Pt layer thickness of 0.35 nm. Finally, the resulting optimized layer stack of the basic motif is realized and provided in Figure 4a. The resulting magnetization reversal of disc-shaped particles consisting of that motif suspended in DI water is shown in Figure 4b. Although there is a slight hysteresis at zero field, the linear magnetization response at low external fields indicates the rotation of these particles in the fluid medium. The switching field can be compared to that obtained from micromagnetic simulation for these particles when still being fixed in an array onto the Si/SiO_2_-substrate. This simulation considers all magnetic interactions such as interlayer exchange interactions, shape and magnetocrystalline anisotropy and dipolar interactions. The underlying magnetic parameters are taken from reference [39]. When fixed to the substrate, there is no magnetization present within the ΔB_ext_ region. Comparing polar Moke curves of the magnetization reversal of particles, consisting of repetitions of the motif see Figure 4 at the lower right, it is apparent that all switching fields are identical because they are purely determined by the interlayer exchange coupling of the Ru-layers. The other aspect of this idea of Cowburn et al., that a multiplication of the motifs also leads to a multiplication of the magnetization and thus also of the magnetic moment of the particles, is shown in Figure 8. These micro-sized particles have nice properties regarding biosensing applications. They do not agglomerate in low external fields and can independently be tuned with respect to switching field and magnetic moment. Thus, they can be applied as a magnetic marker as well as a transmitter of torque from an applied field. The e-beam lithography strategy used here is based on depositing multilayered systems directly onto resist pillars, which release the particles by dissolving them in a solvent. For layered systems that require a suitable crystalline substrate as a structure trigger for epitaxial growth, a different strategy of electron beam lithography should be chosen.

### 3.2. Martensitic Transformation in a Single Elliptical Disc-Shaped Heusler Ni_50_Mn_32.5_Ga_17.5_ Particle

In the second example, the martensitic transformation of the ferromagnetic shape memory Heusler alloy Ni_50_Mn_32.5_Ga_17.5_ [41,42] of a 60 nm thick layer is transferred into free particles. The composition has been chosen in such a way that the martensitic transformation starting at 398 K is completed at room temperature (300 K) and a purely martensitic structure is present [43]. This would change back into a cubic austenite phase during heating. The retransformation is completely finished at 409 K. The austenitic phase for this off-stoichiometric Heusler alloy is paramagnetic and will become ferromagnetic when undergoing the martensitic transformation, thus, at room temperature, it is ferromagnetic.

The 60 nm Heusler alloyed layer is produced by magnetron sputtering. At 500 °C, the desired composition of the layer is adjusted by co-sputtering from a MnGa-composite target and one elemental Ni- and Mn-target each. 

**Figure 5:**
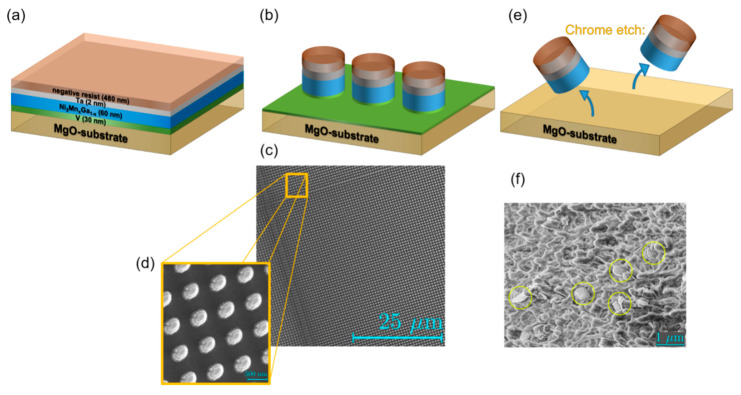
Complete workflow from the layer towards free Ni_50_Mn_32.5_Ga_17.5_ Heusler particles [41]. (**a**) Layer structure with a two nm thick Ta-layer, which protects the Heusler layer from oxidation and the already applied resist layer; (**b**) Multilayered pillars just before etching; (**c**) Scanning electron microscope (SEM) image of these pillars enlarged in (**d**); (**e**) Etching of the V-layer of each pillar and releasing them from the substrate; (**f**) REM image of the filter paper with particles (circled in yellow).

As substrate MgO with (001)-orientation was chosen, on which 30 nm vanadium (V) was deposited as buffer layer. The V takes on two important tasks. Firstly, it conveys the lattice structure of the MgO substrate to the Heusler layer, and secondly, it serves as sacrificial material when the particles are released from the substrate. Vanadium grows epitaxially in [001] direction on MgO, whereupon this Heusler alloy, in turn, grows epitaxially due to the small lattice mismatch [44]. After the sputtering process, the layer is structured into particles by means of several lithography steps. The resist used is a negative resist AR-N7520.18. Spin-coating at 5000 revolutions per minute, it has an approximate thickness of 480 nm. After tempering for 2 min at 85 °C, the resist is exposed and developed in AR-300.47 for 8 min before washing with distilled water to stop the development. Using Ar+ ions, the sample is then etched until pillars consisting of the layer structure are formed. Using the acid Chrome Etch, the next step is to remove the residue caused by the Ar+-etching. By dripping on the acid, the V-layer is removed and the particles are released from the substrate. The particles are then rinsed with ethanol into a filter paper, which is placed under the substrate as a collection tray for the particles. The entire workflow is illustrated in Figure 5.

**Figure 6:**
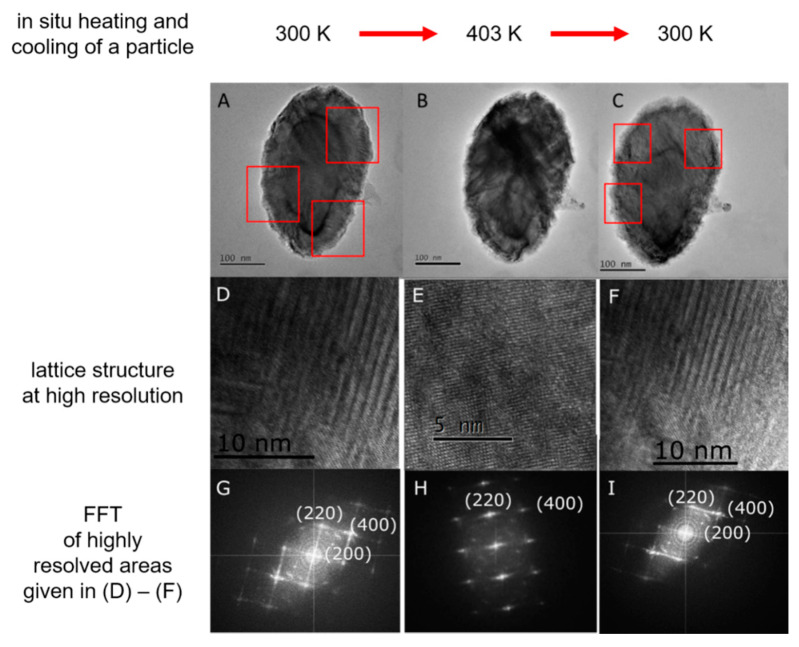
Martensitic transformation in a single elliptical disc-shaped Heusler Ni_50_Mn_32.5_Ga_17.5_ particle, which is about 377 nm long, 234 nm wide and only 60 nm thick [42]. (**Upper row**) TEM pictures (**A**–**C**) of the morphology of the same Heusler Ni_50_Mn_32.5_Ga_17.5_ particle upon temperature change. Size and form of the particle are not changing; (**Middle row**) High-resolution transmission electron microscopy (HRTEM)images of the corresponding lattice changes. (**D**,**F**) clearly show the modulated martensite lattice structure, whereas (**E**) is representative for the cubic austenitic lattice structure; **(Lower row)** Corresponding fast Fourier transforms (FFT) of the highly resolved areas provided in (**D**–**F**). The streaks seeing in (**G**,**I**) confirm the modulated martensite. (**H**) Shows the FFT of a cubic structure.

To prove that the individual particle undergoes the martensitic transition, heating and cooling cycles were performed in a resolution JEOL FS 2200 (JEOL, Tokyo, Japan) employing a dedicated heating TEM holder. These sequences are provided in Figure 6. This proves that the transfer of even a delicate lattice transformation trigged by a distinct temperature cycle from a layered system into a single particle is feasible. 

This gives us hope that such lattice transformations in nanoparticles will open up new applications in biosensor technology. As shown in Figure 6, the volume change associated with the martensitic transformation is very small and is therefore not noticeable by a change in the morphology of the particle. In the future, we, therefore, want to investigate Heusler phases, which are characterized by large volume changes during martensitic transformation [45]. These, in turn, will also lead to a change in the morphology of the corresponding particles. For particles in a microfluidic environment, this is equivalent to a change in their hydrodynamic radius. This opens up the possibility to employ external stimuli, such as a magnetic field, a pressure difference or a temperature difference for switching the hydrodynamic radii of particles in microfluidic devices. Since the magnetic field stabilizes the ferromagnetic phase during a martensitic transformation, the Heusler alloys we will explore further should have a strong ferromagnetic behavior of the austenitic phase, in addition. This would allow us to find new solutions for the separation of biomolecules labeled with these particles, enhancing the typical biosensoric workflow of transport, selection and detection.

### 3.3. Nano-Cylinders from Co_2_MnSi Heusler Compound

**Figure 7:**
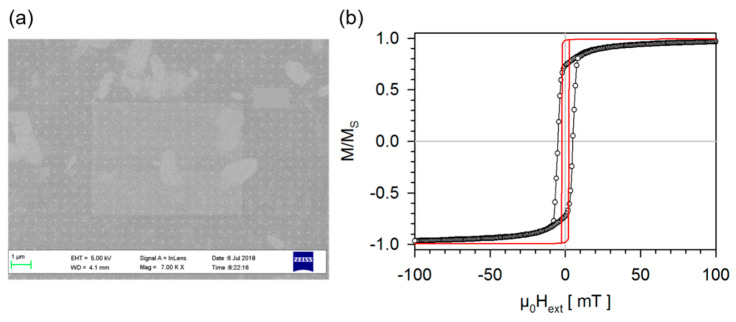
In-plane magnetization reversal of an array of Co_2_MnSi Heusler nano-cylinders. (**a**) REM image of an array of Co_2_MnSi Heusler nano-cylinders during preparation at a stage before the Ar+-ion beam milling is performed. Thus, the Co_2_MnSi Heusler nano-cylinders are capped with Ta. The mean diameter of the nano-cylinders are (46.5 ± 2.7) nm, their height should be 45 nm. (**b**) In black the normalized magnetization of these particles measured by utilizing an alternating gradient magnetometer. In red a micromagnetic calculation of one Co_2_MnSi Heusler nano-cylinder employing the quasistatic micromagnetic simulation package MicroMagus version 7.1. The magnetic parameters are taken from Figure 1. The closest match with the experimental data is found when using a cylinder height of 43.5 nm for these simulations.

The third example now leads to a fabrication variant for small nanoparticles. Strategically, the combination of a positive resist with an Al hard mask is used for electron beam lithography. In detail, the entire manufacturing process is as follows:

Arrays of nano-cylinders from Co_2_MnSi Heusler compound thin films were prepared by e-beam lithography and subsequent Ar+-ion beam milling. The thin films are produced by DC magnetron sputter deposition inside a Leybold Systems Clab 600 cluster tool system with a base pressure better than 2 × 10^−7^ mbar. An amount of 45 nm of Co_2_MnSi was deposited from a 4″ composite target with the same stoichiometry on Si (001) substrates. A 30 nm V seed layer is grown prior to the deposition of the Heusler film. As in the second example, the V-layer takes on a double role. It conveys the lattice structure of the MgO substrate to the Heusler layer and it serves as sacrificial material when the particles are finally released from the substrate. The samples are finally capped by a 2 nm thick Ru layer to prevent oxidation. No heat treatment is performed during or after the deposition, resulting in polycrystalline thin films with a preferred [011]-texture in the out-of-plane direction for both V and Co_2_MnSi. To remove any organic contaminants on the sample surfaces, they were cleaned by ultrasonic treatment for 5 min in isopropanol, followed by 2 min in ethanol, and finally, 2 min in DI water and dried under a nitrogen flow. A single layer of AR-P 617.03 positive resist from the Allresist company is spin-coated on the sample with 6000 rpm for 30 s and soft baked at 210 °C for 20 min on a hot plate. Exposure is performed inside a Carl Zeiss 1520 scanning electron microscope equipped with a Raith lithography system with a dose of 0.6$\;\mu \mathrm{As}/{\mathrm{cm}}^{2}$. To make the production of the particle array efficient, a rectangle is drawn that completely covers each writing area. The step size is set to 500 nm in the pattern generator, resulting in an exposure of dots evenly spaced at 500 nm. After exposure, the samples are developed for 2 min in AR-P 600-50, immersed in AR-P 600-60 stopper bath and blown dry under a nitrogen flow. Additionally, a post bake at 120 °C for 2 min on a hot plate is performed. A metal mask for the exposed areas is produced by depositing a 58 nm Ta layer in a custom build sputter chamber on the samples. Its thickness is adjusted so that after the milling of the Ar+-ion beam, the Ta metal mask is removed and the unprotected areas of the Co2MnSi layer appear. Removal of the resist is accomplished in an ultrasonic treatment in NMP (N-Methyl-2-pyrrolidone) for 15 min followed by a 5 min cleaning step in ethanol. Finally, Ar+-ion beam milling is performed on the samples in a custom build system, which is constantly monitored by secondary ion mass spectrometry, until the V seed layer is reached. In a final step, Chrome etch can be applied to remove the V-layer of each pillar and releasing Co_2_MnSi Heusler the nano-cylinders 46.5 nm in diameter and 45 nm in height from the substrate. The in-plane magnetization reversal of such an array of Co_2_MnSi Heusler nano-cylinders is provided in Figure 7.

Indeed, this top-down approach is capable of producing relatively small Heusler nanoparticles, which will benefit from the intrinsic magnetic properties of Co_2_MnSi.

Even the micromagnetic calculations presented in Figure 7b are comparable with the achieved experimental data. The experimentally determined magnetization curve shows a slight hysteresis which is due to overcoming the superparamagnetic limit that is provided for a cylindrical particle assuming a stable state over one year according to Equation (3) as:

(3) $$L_{spm}^{cylinder}=\sqrt{\frac{152Tk_{B}}{\pi Kh}},$$

with *T* = 300 K, *K* the magnetocrystalline anisotropy of Co_2_MnSi, see above, *k_B_* the Boltzmann constant and *h* the height of the cylindrical particle. Using the result of the simulation for *h* = 43.5 nm, the superparamagnetic limit is calculated to be 39.93 nm which is less than the diameter of Co_2_MnSi Heusler nano-cylinders of about 46 nm. The resulting coercivity of 5.04 mT is in the range of the anisotropy field of 3.16 mT for the in-plane measurement. Hence, the measured and expected magnetic data of theCo_2_MnSi Heusler nano-cylinders do reasonably match.

With regard to the application of nanoparticles in biosensor technology, these nanoparticles are of the desired size for labeling of selected polysomes mentioned above.

### 3.4. Validating the Potential of These Particles Employing the Concept of a Magnetic On–Off Ratchet

**Figure 8:**
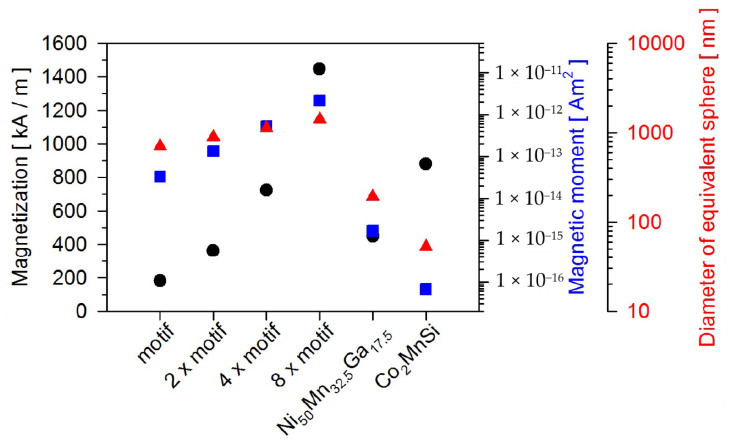
Resulting magnetization in black and magnetic moment in blue determined from the structural data of the particles produced by the top-down approach. Comparing the evolution of the magnetization of the particles when repeating the motif presented in Section 3.1 indicates their gain in magnetization as a function of the repetition. To be able to estimate the stray field interaction of these particles with a magnetoresistive sensor, the diameter of spheres with the equivalent volume of these particles is calculated and shown in red.

**Figure 9:**
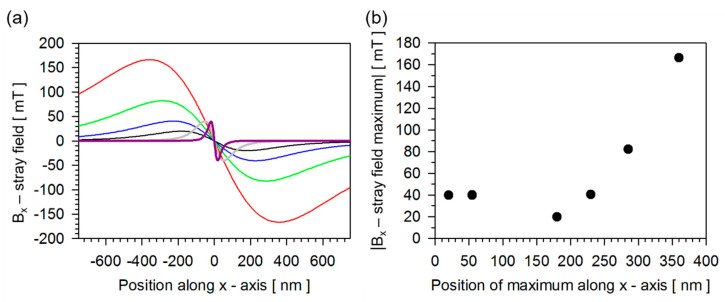
B_x_ —component of the dipolar stray field range of the particles with magnetic parameters provided in Figure 8: in black—motif, in blue 2xmotif, in green 4xmotif, in red 8xmotif, in grey Ni_50_Mn_32.5_Ga_17.5_ and in dark magenta Co_2_MnSi; (**a**) B_x_ component of the stray field along the *x*-direction of a particle positioned 10 nm above the surface of a magnetoresistive sensor; (**b**) Position and amplitude of the maxima of the B_x_ component of the stray fields imaged in (**a**). The dipolar stray field has been calculated applying [47] assuming a fully saturated particle in *z*-direction.

In the last part, the question will be answered which properties these particles produced by the top-down approach actually have in biosensor technology. If one defines the task of magnetic particles as carriers of biomolecules, then with regard to biosensor technology, the particles take over three essential tasks in a microfluidic environment, the magnetically-driven transport of biomolecules, the magnetically initiated separation of biomolecules and causing the detection of biomolecules. Essential for magnetically-driven transport is the magnetic moment of the particles, by which the movement of the particle with and without biomolecule loading in magnetic stray field landscapes can be realized. Figure 8 summarizes the magnetic properties of these particles produced by the top-down approach. The detection of biomolecules labelled with the magnetic particles is determined by the interaction of the particles’ stray magnetic fields with a magnetoresistive sensor and is visualized in Figure 9. Assuming that all particles are positioned 10 nm above the surface of a magnetoresistive sensor, magnetized in *z*-direction, the in-plane interaction range of the corresponding dipolar stray fields of all particles is demonstrated with reasonable amplitudes. Simultaneously, it becomes evident that the geometry of the sensors would necessarily be adjusted to the interaction range of the regarded particles. Due to the sufficiently high magnetic moments of all the different particles, they can be transported to the sensor in inhomogeneous magnetic fields and concentrated there. The sorting of magnetic particles is possible with a magnetic field in combination with another effect, such as diffusion or hydrodynamic flow. It is precisely this interplay of diffusion and magnetic fields that is exploited in the magnetic on–off ratchet [22], which represents a system capable of converting non-directional thermal fluctuation into directional motion. The concept of the magnetic ratchet is used here to simulate size dependent on the influence of the magnetic potential on the mobility of the particles. Whereas the development of this simulation, which is an extension of that used in [22], is derived in [46], here we elaborate on a major result pictured in Figure 11.

**Figure 10:**
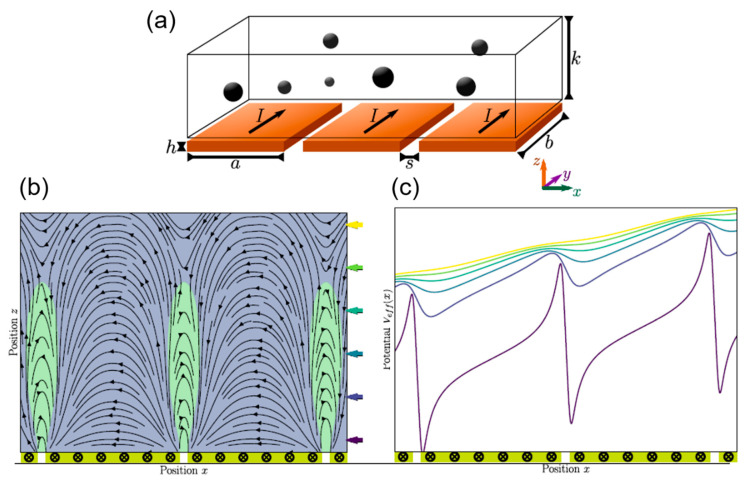
Magnetic on–off ratchet according to [22]; (**a**) A microfluidic channel of width b and height k, under which a series of conductors of width a and thickness h are located at a distance s. Experimentally, a fixed channel height can easily be realized by closing the channel in *z*-direction using a glass cover; (**b**) x-z cross-section through the force field (F_x_, F_z_), represented by the streamlines, with F_x_ > 0 and F_x_ < 0 (**c**) Line section at constant height through the potential landscape, the z-coordinate corresponds to the colored marking by the arrows in (**b**). The tilting of the potential is only present for very short ratchets, i.e., only a few current paths, and disappears when the ratchet consists of many current paths.

The magnetic on–off ratchet shown in Figure 10a consist of a microfluidic channel, which is mounted on a series of conductor paths. In addition, the magnetic moments of particles in the channel are aligned with a modest homogeneous magnetic field in the *z*-direction.

This field provides both a simplified interaction between the particles and the magnetic field and ensures that the particles are distributed among each other as widely as possible as long as their concentration is low, hence neglecting particle–particle interaction. If a current now flows through the conductor tracks (on-state with τ_on_), a magnetic ratchet potential emerges, see Figure 10c. The particles follow that potential and accumulate, lined up in the *y*-direction at the edges of the tracks. When the power is switched off, the particles move freely (off-state with τ_off_) under the influence of Brownian motion. Through the periodic change, between on- and off-state, such a directed motion is created. By reversing the current flow, the particle flow is also reversed. The force field (Fx, Fz), represented by the streamlines, and the potential landscape is calculated by a Finite Element Method already used in [22].

**Figure 11:**
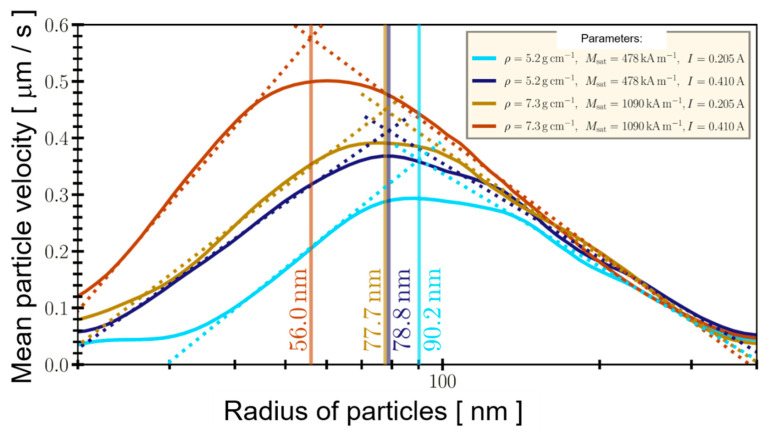
Simulated mean particle velocity as a function of the radius of the particles for Magnetite (ρ = 5.2 g/cm, M_s_ = 478 kA/m) and Co_2_FeSi (ρ = 7.3 g/cm, M_s_ = 1090 kA/m). Geometry of the ratchet with six conduction paths, for each path: a = 41 μm, h = 0.1 μm, b = 200 μm, s = 2.5 μm, k = 20 μm. Two current flows through each conductor are tested, I = 0.205 A and I = 0.410 A. The switching times are τ_on_ = 11.5 s and τ_off_ = 3.5 s. The dotted lines serve as guidelines to the eyes indicating the positions of the maxima of the mean particle velocities.

With this concept, we want to estimate the size dependence of the mean particle velocity in the ratchet system. Physically, one expects an optimal particle radius for a fixed ratchet geometry and each parameter set of particle size, magnetic moment of the particles and applied current, since the magnetic moment is $m\propto R^{3}$ and hence the force $F\propto m\propto R^{3}$. This should result in a quadratic dependence of the expected speed of the particles on their radius since $v_{Stokes}\propto F/R\propto R^{2}$. On the other hand, the diffusive transport in the regime of Brownian motion via the diffusion constant D is related as $D\propto R^{-1}$. Figure 11 shows the results of such variation of the particle radius comparing Magnetite with Co_2_FeSi, a Heusler very similar to Co_2_MnSi.

Clearly, Figure 11 shows the optimal particle radii for the two materials at different current conditions. In comparison to magnetite, it is evident that the Heusler alloy is a good material choice here, since for the two selected current values, the Heusler particles with a diameter of 112 nm could be transported in such a ratchet at 0.5 μm/s and with a diameter of 155 nm at 0.39 μm/s. If these proportions are transferred to the equivalent diameters shown in Figure 8 for spheres of the three examples of particles produced by the top-down approach, only the Co_2_MnSi particles remain, which could be moved at about 0.2 μm/s in the ratchet. All other particles are already too large. Thus, the results can now be discussed against the background of biosensor technology.

## 4. Discussion

Concerning the preparation of particles, it turned out that the experimental challenge for all three examples is the release of particles or nanoparticles fixed on different substrates into freely moving particles. Although this process worked in all cases, the yield was sobering. This defines the task of improving this yield significantly in the future.

Size-wise, the Co_2_MnSi nanoparticles can be used in a microfluidic device such as the magnetic on–off ratchet. Nevertheless, these particles are cylindrical and hence their proper hydrodynamic radius is different from that of a sphere and should be taken into account. The same argument is valid for the elliptical plate-like Ni_50_Mn_32.5_Ga_17.5_particles, which are about a factor of two too big to be transported by the on–off ratchet. However, this is not a hurdle in principle, as the current range and consequently the magnetic potential of the magnetic on–off ratchet can easily be enhanced by increasing the thickness of conduction paths of the ratchet by a factor of 20. Moreover, the pulsed current could be employed as well to enhance this further. The underlying switching strategy of the ratchet can also be changed. While particle transport for the ratchets relies on diffusion, a deterministic drift by switching currents in such a way that the particles always “slide down” the long slope of the potential in Figure 10c is also possible. This is achieved by sending a current only through the even conduction paths during the first half-period. During this phase, the particles move towards the minima of even conduction paths. In the second half-period, the even wires are switched off and the odd ones are turned on instead, which effectively shifts the potential by the distance between neighboring conduction paths in space. Consequently, the particles that previously aggregated at the minima of the even conduction paths now sit right on the long slopes towards the minima of the odd conduction paths and thus continue their drift in this direction.

In summary, there are still many degrees of freedom to adjust the proper magnetic ratchet to the size properties of the particles fabricated in a top-down approach, which can be explored in the future.

Recently, a new bottom-up preparation method for magnetic nanoparticles and complex structures has attracted attention because it is based on focused electron beam induced deposition [48], a direct-write method for the fabrication of nanostructures whose lateral resolution rivals that of advanced electron-beam lithography. It is based on focused ion beam technology and is capable of using metalorganic precursors such that Co_2_(OH)_8_ to realize Co-nanoparticles by decomposing the precursor.

Thus, the race between bottom-up and top-down is yet undecided for the production of magnetic nanoparticles for application in biosensorics, remaining exciting and scientifically fruitful.

## 5. Conclusions

Starting from investigating the intrinsic magnetic properties of different magnetic phases, we reviewed the magnetic properties of nanoparticles for biosensing. Employing different e-beam lithography strategies, we demonstrated thereafter that three different particle systems, tunable perpendicularly magnetized synthetic antiferromagnets, martensitic transformation in a single elliptical disc-shaped Heusler Ni_50_Mn_32.5_Ga_17.5_ particle and nano-cylinders from the Co_2_MnSi Heusler compound, were fabricated via a top-down approach ranging from the μm- towards nm-scale. Based on our calculations, as well as on the comparison of experimental data, we identified nano-cylinders from the Co_2_MnSi Heusler compound as the most suitable particle system for biosensing application. (1) It has a large superparamagnetic limit of 39.93 nm. (2) The resulting dipolar stray interaction with a magnetoresistive sensor, compared with Figure 9a, is excellent. It will ensure a large amount of particle detection due to a very small interaction range in 2D, compared with Figure 9b. (3) The very large susceptibility could be theoretically shown and experimentally validated, see Figure 7b. (4) Finally, with the concept of a magnetic on–off ratchet, we demonstrated that Co_2_FeSi, which is magnetically very close to Co_2_MnSi, is most suitable for magnetically-driven transport in a microfluidic channel and superior to Magnetite. Therefore, we can conclude the same for Co2MnSi. In addition, options are also discussed for improving the magnetic ratchet for larger particles.

## References

[1] Baibich M.N., Broto J.M., Fert A., Van Dau F.N., Petroff F., Etienne P., Creuzet G., Friederich A., Chazelas J. (1988). Giant Magnetoresistance of (001)Fe/(001)Cr Magnetic Superlattices. Phys. Rev. Lett..

[2] Binasch G., Grunberg P., Saurenbach F., Zinn W. (1989). Enhanced magnetoresistance in layered magnetic structures with antiferromagnetic interlayer exchange. Phys. Rev. B.

[3] Baselt D.R., Lee G.U., Natesan M., Metzger S.W., Sheehan P.E., Colton R.J. (1998). A biosensor based on magnetoresistance technology. Biosens. Bioelectron..

[4] Tondra M., Porter M., Lipert R.J. (2000). Model for detection of immobilized superparamagnetic nanosphere assay labels using giant magnetoresistive sensors. J. Vac. Sci. Technol. A.

[5] Lagae L., Wirix-Speetjens R., Das J., Graham D., Ferreira H.A., Freitas P., Borghs G., De Boeck J. (2002). On-chip manipulation and magnetization assessment of magnetic bead ensembles by integrated spin-valve sensors. J. Appl. Phys..

[6] Graham D., Ferreira H.A., Bernardo J., Freitas P., Cabral J.M.S. (2002). Single magnetic microsphere placement and detection on-chip using current line designs with integrated spin valve sensors: Biotechnological applications. J. Appl. Phys..

[7] Schotter J., Kamp P., Becker A., Pühler A., Reiss G., Brückl H. (2004). Comparison of a prototype magnetoresistive biosensor to standard fluorescent DNA detection. Biosens. Bioelectron..

[8] Chan C.P., Cheung Y.-C., Renneberg R., Seydack M. (2007). New Trends in Immunoassays. Adv. Biochem. Eng. Biotechnol..

[9] Mattiasson B., Teeparuksapun K., Hedström M. (2010). Immunochemical binding assays for detection and quantification of trace impurities in biotechnological production. Trends Biotechnol..

[10] Graham D., Ferreira H.A., Freitas P., Cabral J.M.S. (2003). High sensitivity detection of molecular recognition using magnetically labelled biomolecules and magnetoresistive sensors. Biosens. Bioelectron..

[11] Freitas P., Cardoso F., Martins V.C., Martins S.A.M., Loureiro J., Amaral J., Chaves R.C., Cardoso S., Fonseca L.P., Sebastião A.M. (2012). Spintronic platforms for biomedical applications. Lab Chip.

[12] Schrittwieser S., Pelaz B., Parak W.J., Lentijo-Mozo S., Soulantica K., Dieckhoff J., Ludwig F., Guenther A., Tschöpe A., Schotter J. (2016). Homogeneous Biosensing Based on Magnetic Particle Labels. Sensors.

[13] Reiss G., Brueckl H., Hütten A., Schotter J., Brzeska M., Panhorst M., Sudfeld D., Becker A., Kamp P.B., Puehler A. (2005). Magnetoresistive sensors and magnetic nanoparticles for biotechnology. J. Mater. Res..

[14] Hütten A., Sudfeld D., Ennen I., Reiss G., Hachmann W., Heinzmann U., Wojczykowski K., Jutzi P., Saikaly W., Thomas G. (2004). New magnetic nanoparticles for biotechnology. J. Biotechnol..

[15] Lu A.-H., Salabas E.L., Schuth F. (2007). Magnetische Nanopartikel: Synthese, Stabilisierung, Funktionalisierung und Anwendung. Angew. Chem..

[16] Van Waeyenberge B., Puzic A., Stoll H., Chou K.W., Tyliszczak T., Hertel R., Faehnle M., Brueckl H., Rott K., Reiss G. (2006). Magnetic vortex core reversal by excitation with short bursts of an alternating field. Nature.

[17] Mühlbauer S., Binz B., Jonietz F., Pfleiderer C., Rosch A., Neubauer A., Georgii R., Boni P. (2009). Skyrmion Lattice in a Chiral Magnet. Science.

[18] Zhu T., Chen P., Zhang Q., Yu R.C., Liu B.-G. (2014). Giant linear anomalous Hall effect in the perpendicular CoFeB thin films. Appl. Phys. Lett..

[19] Kiyohara N., Tomita T., Nakatsuji S. (2016). Giant Anomalous Hall Effect in the Chiral Antiferromagnet Mn3Ge. Phys. Rev. Appl..

[20] Kim K.W., Reddy V., Torati S.R., Hu X.H., Sandhu A., Kim C.G. (2015). On-chip magnetometer for characterization of superparamagnetic nanoparticles. Lab Chip.

[21] Cubero D., Renzoni F. (2016). Brownian Ratchets: From Statistical Physics to Bio and Nano-Motors.

[22] Auge A., Weddemann A., Wittbracht F., Hütten A. (2009). Magnetic ratchet for biotechnological applications. Appl. Phys. Lett..

[23] Holzinger D., Lengemann D., Göllner F., Engel D., Ehresmann A. (2012). Controlled movement of superparamagnetic bead rows for microfluid mixing. Appl. Phys. Lett..

[24] Ueltzhöffer T., Streubel R., Koch I., Holzinger D., Makarov D., Schmidt O.G., Ehresmann A. (2016). Magnetically Patterned Rolled-Up Exchange Bias Tubes: A Paternoster for Superparamagnetic Beads. ACS Nano.

[25] Reiss G., Hütten A. (2005). Magnetic nanoparticles: Applications beyond data storage. Nat. Mater..

[26] Tietze R., Zaloga J., Unterweger H., Lyer S., Friedrich R.P., Janko C., Pöttler M., Dürr S., Alexiou C. (2015). Magnetic nanoparticle-based drug delivery for cancer therapy. Biochem. Biophys. Res. Commun..

[27] Angelakeris M. (2017). Magnetic nanoparticles: A multifunctional vehicle for modern theranostics. Biochim. Biophys. Acta.

[28] Su Y., Dostmann W.R., Herberg F.W., Durick K., Xuong N.H., Eyck L.T., Taylor S.S., Varughese K.I. (1995). Regulatory subunit of protein kinase A: Structure of deletion mutant with cAMP binding domains. Science.

[29] Wegener M., Ennen I., Walhorn V., Anselmetti D., Hütten A., Dietz K.-J. (2019). Magnetic Tracking of Protein Synthesis in Microfluidic Environments-Challenges and Perspectives. Nanomaterials.

[30] Coey J.M.D. (2010). Magnetism and Magnetic Materials.

[31] Kuang F.G., Kuang X.Y., Kang S.Y. (2014). A Systematic Investigation on Magnetism and Phase Stability of Cobalt. Z. Nat. A.

[32] De la Peña O’Shea V.A., Moreira I.D.P., Roldán A., Illas F. (2010). Electronic and magnetic structure of bulk cobalt: The α, β, and ε-phases from density functional theory calculations. J. Chem. Phys..

[33] Kämmerer S., Thomas A., Hütten A., Reiss G. (2004). Co 2 Mn Si Heusler alloy as magnetic electrodes in magnetic tunnel junctions. Appl. Phys. Lett..

[34] Betancourt-Cantera J., Jesús F.S.-D., Bolarín-Miró A., Torres-Villaseñor G., Betancourt-Cantera L. (2019). Magnetic properties and crystal structure of elemental cobalt powder modified by high-energy ball milling. J. Mater. Res. Technol..

[35] Grob D.T., Wise N., Oduwole O., Sheard S. (2018). Magnetic susceptibility characterisation of superparamagnetic microspheres. J. Magn. Magn. Mater..

[36] Graf T., Felser C., Parkin S.S.P. (2011). Simple rules for the understanding of Heusler compounds. Prog. Solid State Chem..

[37] Stearns M.B., Cheng Y. (1994). Determination of para- and ferromagnetic components of magnetization and magnetoresistance of granular Co/Ag films (invited). J. Appl. Phys..

[38] Dreyer A., Peter M., Mattay J., Hütten A., Jutzi P. (2012). Ionic Additives and Weak Magnetic Fields in the Thermo-Decomposition of Dicobalt Octacarbonyl: Tools for the Morphology Control of Cobalt Nanoparticles. Eur. J. Inorg. Chem..

[39] Lavrijsen R., Fernández-Pacheco A., Petit D., Mansell R., Lee J.H., Cowburn R. (2012). Tuning the interlayer exchange coupling between single perpendicularly magnetized CoFeB layers. Appl. Phys. Lett..

[40] Vemulkar T., Mansell R., Petit D.C.M.C., Cowburn R.P., Lesniak M.S. (2015). Highly tunable perpendicularly magnetized synthetic antiferromagnets for biotechnology applications. Appl. Phys. Lett..

[41] Gottschalk M. (2017). Anwendungen der Ionen- & Elektronenmikroskopie im Grenzgebiet Zwischen Nanostrukturphysik und Biologie. Ph.D. Thesis.

[42] Haase M. (2018). Martensitische Nanopartikel. Ph.D. Thesis.

[43] Xu X., Nagasako M., Ito W., Umetsu R.Y., Kanomata T., Kainuma R. (2013). Magnetic properties and phase diagram of Ni50Mn50−xGax ferromagnetic shape memory alloys. Acta Mater..

[44] Teichert N. (2016). Shape Memory Heusler Alloys for Thin Film Applications. Ph.D. Thesis.

[45] Wei Z., Liu E., Li Y., Han X.L., Du Z.W., Luo H., Liu G., Xi X.-K., Zhang H., Wang W.H. (2016). Magnetostructural martensitic transformations with large volume changes and magneto-strains in all-d-metal Heusler alloys. Appl. Phys. Lett..

[46] Kappe D. (2020). Simulationen der Mikrostruktur und Dynamik von Nanopartikeln im Kontext von Hydrodynamik und Magnetoresistivem Transport. Ph.D. Thesis.

[47] Craik D., Brown R.W. (1996). Magnetism: Principles and Applications. Phys. Today.

[48] Huth M., Porrati F., Dobrovolskiy O.V. (2018). Focused electron beam induced deposition meets materials science. Microelectron. Eng..

